# Conditioned medium from induced pluripotent stem cell-derived mesenchymal stem cells accelerates cutaneous wound healing through enhanced angiogenesis

**DOI:** 10.1186/s13287-021-02366-x

**Published:** 2021-05-20

**Authors:** Xiaoting Liang, Fang Lin, Yue Ding, Yuelin Zhang, Mimi Li, Xiaohui Zhou, Qingshu Meng, Xiaoxue Ma, Lu Wei, Huimin Fan, Zhongmin Liu

**Affiliations:** 1 Institute for Regenerative Medicine, Shanghai East Hospital, School of Life Sciences and Technology, Tongji University, Shanghai, Peoples Republic of China; 2 Research Center for Translational Medicine, Shanghai East Hospital, School of Medicine, Tongji University, Shanghai, Peoples Republic of China; 3 Laboratory of Arrhythmias, Ministry of Education of China, Shanghai East Hospital, Tongji University, Shanghai, Peoples Republic of China; 4 Department of Organ Transplantation, Changzheng Hospital, Second Military Medical University, Shanghai, Peoples Republic of China; 5 Department of Emergency, Guangdong General Hospital, Guangdong Academy of Medical Science, Guangzhou, Peoples Republic of China; 6 Department of Cardiovascular Surgery, Shanghai East Hospital, Tongji University, Shanghai, Peoples Republic of China

**Keywords:** Induced pluripotent stem cell-derived mesenchymal stem cells, Conditioned medium, Wound healing, Mitochondria dysfunction

## Abstract

**Background:**

Mesenchymal stem cells (MSCs) can improve cutaneous wound healing via the secretion of growth factors. However, the therapeutic efficacy of MSCs varies depending upon their source. Induced pluripotent stem cells are emerging as a promising source of MSCs with the potential to overcome several limitations of adult MSCs. This study compared the effectiveness of conditioned medium of MSCs derived from induced pluripotent stem cells (iMSC-CdM) with that derived from umbilical cord MSCs (uMSC-CdM) in a mouse cutaneous wound healing model. We also investigated the mechanisms of protection.

**Methods:**

The iMSC-CdM or uMSC-CdM were topically applied to mice cutaneous wound model. The recovery rate, scar formation, inflammation and angiogenesis were measured. We compared angiogenesis cytokine expression between iMSC-CdM and uMSC-CdM and their protective effects on human umbilical vein endothelial cells (HUVECs) under H_2_O_2_-induced injury. The effects of iMSC-CdM on energy metabolism, mitochondria fragmentation and apoptosis were measured.

**Results:**

Topical application of iMSC-CdM was superior to the uMSC-CdM in accelerating wound closure and enhancing angiogenesis. Expression levels of angiogenetic cytokines were higher in iMSC-CdM than they were in uMSC-CdM. The iMSC-CdM protected HUVECs from H_2_O_2_ induced injury more effectively than uMSC-CdM did. Administration of iMSC-CdM stimulated HUVEC proliferation, tube formation and energy metabolism via the ERK pathway. Mechanistically, iMSC-CdM inhibited H_2_O_2_-induced mitochondrial fragmentation and apoptosis of HUVECs.

**Conclusion:**

Collectively, these findings indicate that iMSC-CdM is more effective than uMSC-CdM in treating cutaneous wounds, and in this way, iMSC-CdM may serve as a more constant and sustainable source for cell-free therapeutic approach.

**Graphical abstract:**

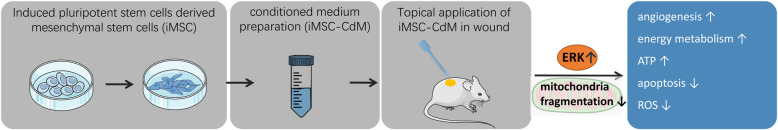

**Supplementary Information:**

The online version contains supplementary material available at 10.1186/s13287-021-02366-x.

## Introduction

The skin forms a barrier between the inside and the outside of an organism. Injury of the skin may impair its barrier function and expose the inside tissues to pathogens and mechanical damage. In cutaneous wound healing, damaged tissue is repaired through a regenerative process orchestrated by multiple biological pathways [1]. Various therapeutic approaches are available to treat wounds, but current wound-care practices achieve limited effectiveness. Regenerative medicine emerges as an alternative intervention to improve the healing process. Mesenchymal stem cells (MSCs), also known as multipotent mesenchymal stromal cells, are characterized by the capacity for self-renewal and multilineage differentiation. MSCs are one of the most promising tools for regenerative medicine and offer significant therapeutic potential in various diseases, including wound closure. However, more research is needed to determine the ideal sources of therapeutic MSCs and methods for MSC delivery.

The efficacy of MSCs varies depending on their source. Hsieh et al. reported that Wharton jelly MSCs induced better microvasculature formation and cell migration than bone marrow MSCs [2]. In the diabetic mouse wound model, Kim et al. determined that amniotic MSCs significantly enhanced the rate of fibroblast wound closure and re-epithelialization versus adipose-derived MSCs [3]. However, there are limitations associated with MSCs derived from adult tissues, including finite cell proliferative capacity, alterations in phenotype and differentiation potential after long-term culture [4]. Therefore, MSCs derived from induced pluripotent stem cells (iMSCs) are emerging as an attractive option, because during the reprogramming process, iMSCs acquire a rejuvenation signature and exhibit improved cellular vitality such as survival, proliferation, and differentiation potential [5]. iMSCs can be expanded up to 120 passages without showing senescent signatures [6] and have therapeutic potential in the regeneration of blood vessels and periodontal, liver, heart, and lung tissues [6,10].

Delivery method is an essential contributing factor in MSC therapy. In many studies of cutaneous wound healing, MSCs were intradermally injected into or around the wound area. Although this method has improved wound healing, poor cell engraftment efficiency limits therapeutic benefits [11]. MSCs exert their therapeutic action mainly by paracrine secretion, such as growth factors, cytokines, chemokines and extracellular microvesicles, into their surroundings. For this reason, cell-free MSC-based approaches have been applied to treat cutaneous wounds. Conditioned medium of Whartons jelly-derived MSCs was more beneficial in the recovery of radiation-induced skin wounds than epidermal growth factor (EGF) [12]. Microencapsulated MSCs attenuated immune cell infiltration, facilitated wound closure and accelerated re-epithelialization [13]. However, the treatment effects of iMSC on cutaneous wound healing have not yet been determined.

The reduced angiogenesis ability is acknowledged as a main contributor to the dysfunctional healing response [14]. Mitochondrial respiration and function control neoangiogenesis in endothelial cells during wound healing [15, 16]. As highly dynamic organelles, mitochondria can adapt their morphology and function through fusion and fission events in response to environmental change. The fusion process is mediated by mitofusin 1 (MFN1) and mitofusin 2 (MFN2), while fission is accomplished by dynamin-related protein 1 (DRP1). Mutants for mitochondrial fusion and fission proteins fail to close open wounds, indicating the regulation of mitochondrial dynamics is required for wound healing [17]. However, whether iMSC delivered better effects in wound closure through regulating mitochondrial dynamics in endothelial cells remains to be addressed.

In the current study, conditioned medium from iMSCs (iMSC-CdM) and that from umbilical cord MSCs (uMSC-CdM) was topically administered to a mouse cutaneous wound healing model for comparison of their therapeutic efficacies. We revealed that iMSC-CdM was superior to uMSC-CdM in accelerating wound closure with consequent enhanced angiogenesis and regulating the dynamic balance of mitochondria fusion and fission in endothelial cells.

## Materials and methods

### Preparation and characterization of iMSCs and uMSCs

Human iMSCs were differentiated from human induced pluripotent stem cell (hiPSC) line IMR90-iPSCs (WiCell Research Institute, Madison, WI, USA) as previously described [6, 18]. Briefly, hiPSCs were cultured in MSC differentiation medium which consists of 10% fetal bovine serum (FBS), 5ng/mL basic fibroblast growth factor (bFGF; AF-100-18B, Peprotech), 5ng/mL epidermal growth factor (EGF; AF-100-15, Peprotech) and 55uM 2-mercaptoethanol (Gibco, 21985023). One week later, differentiating iPSCs were purified by sorting for CD24CD105^+^ population using a fluorescence-activated cell sorting system. Human umbilical cord MSCs (uMSCs) were purchased commercially (Saliai, Guangzhou, China). MSCs were cultured with minimum essential medium plus 5% UltraGRO-Advanced GMP cell culture supplement(Helios, HPCFDCGL50). MSCs were characterized by surface marker profiling (CD34, CD45, CD73, CD90 and CD105) and trilineage differentiation (adipogenesis, osteogenesis and chondrogenesis) as previously described [19]. Cells at passage 4 through passage 8 were used for the following experiments.

### Preparation of conditioned medium

Cells at passage 4 through passage 8 were seeded into 10cm culture dishes and allowed to reach 60% to 70% confluence. The medium was then changed to minimum essential medium (5mL), and the cells were cultured for another 24h. Next, the conditioned medium was collected, centrifuged to remove the debris, filtered, and stored at 80C.

### In vivo wound generation and macroscopic examination

Male Balb/C mice, 6 to 8weeks old, were purchased from Vital River (Beijing, China). Mice were randomly assigned to iMSC-CdM, uMSC-CdM, or vehicle control groups (10 in each group). All mice were anesthetized by intraperitoneal injection of 50mg/kg pentobarbital before surgery. A 10-mm-diameter, full-thickness excisional wound was created under sterile surgical conditions. The wounds were covered with a sterile cotton pad followed by topical administration of uMSC-CdM or iMSC-CdM (100L). An equivalent volume of minimum essential medium was administered in the control group. Thirty minutes later, the cotton pad was removed from the wounds. The treatment mentioned above was conducted daily. The epithelial gap was measured every day. The recovery rate was expressed as a percentage of the original wound size with the following formula: (1-remaining wound size/original wound size) 100%. Mice were sacrificed at9days after surgery.

### Immunohistochemical and immunofluorescence analyses

The collected specimens were fixed in 4% paraformaldehyde solution, dehydrated with a series of graded ethanol, and embedded in paraffin. Sections were stained with Masson trichrome and photographed under an optical microscope. Immunofluorescence staining for CD31 (Abcam, ab76533), vimentin (Cell Signaling, 5741), and cytokeratin (Abcam, ab9377) was performed to assess the extent of newly formed microvessels, fibroblasts, and keratinocytes, respectively. Six random fields per section near wound edges were counted with Image-Pro Plus 6 software.

### Cytokine profiling

The skin specimens were lysed with lysis buffer (Cell Signaling, 9803). Proteins were quantified by BCA Protein Assay Kit (Thermo Fisher, 23327). The expression levels of interleukin-6 (IL6), monocyte chemoattractant protein-1 (MCP1), interferon- (IFN), interleukin-10 (IL10), interleukin-12p70 (IL12p70), and tumor necrosis factor-alpha (TNF) were measured with a bead-based analyte detection system (BD Biosciences, 552364) according to the manufacturers instructions. The expression of EGF and vascular endothelial growth factor A (VEGFA) was determined by enzyme-linked immunosorbent assay (ELISA) according to the manufacturers instructions (Multiscience, EK283 & EK293).

### Primary skin fibroblast isolation

Primary skin fibroblasts were isolated as previously described [20]. Briefly, skin tissues were obtained from 1 to 3days old mice. The skin tissues were cut into approximately 1mm pieces and digested with 2mg/mL type I collagenase (Sigma-Aldrich, C0130) for 2h. After washing, the precipitate was resuspended in the culture medium (DMEM/F12 supplemented with 15%FBS) and transferred to a 10-cm dish. The fibroblasts started to exit tissue fragments within 1week.

### Transwell assay

To determine whether uMSC-CdM/iMSC-CdM affected cell mobility, 2 10^4^ skin fibroblasts were seeded on the upper chamber of the transwell apparatus with a pore size of 8m (Corning, 3422). In the lower chamber, uMSC-CdM and iMSC-CdM supplemented with 2% FBS were added as attractants, and basal medium supplemented with 2% FBS was set as the control group. Six hours later, the cells were fixed using 4% paraformaldehyde for 30min. Non-migrated cells were scraped off the upper surface of the membrane with a cotton swab. Migrated cells that remained on the lower surface were stained with 2-(4-Amidinophenyl)-6-indolecarbamidine dihydrochloride (DAPI). There were triplicate wells with 6 random sights for each well for each group.

### Quantitative reverse transcription polymerase chain reaction

Total RNA was extracted from skin specimens with RNeasy Mini Kit (Qiagen, 74124). cDNA was synthesized from 500ng of total RNA with a RevertAid First Strand cDNA Synthesis Kit (Takara, RR0036A). Then, quantitative reverse transcription polymerase chain reaction analysis was performed with Fast SYBR Green Master Mix (4385617) in an ABI QuantStudio 6 Flex System. The relative standard curve method (2^CT^) was used to determine the relative mRNA expression, with glyceraldehyde 3-phosphate dehydrogenase (GAPDH) as the reference gene. The polymerase chain reaction primers used in this study are listed in Table 1. Mitochondrial energy metabolism PCR array (Qiagen, PAHS-008Y) was performed to detect the expression profile of 84 genes associated with mitochondrial respiration, including genes encoding components of the electron transport chain and oxidative phosphorylation complexes. The median cycle threshold value (CT) was uploaded onto the manufacturers web portal (https://geneglobe.qiagen.com/cn/analyze), and the fold change of each gene expression was calculated using the provided software according to the manufacturer's instruction.

**Table 1:** Primer sequence

Gene	Sequence
Mouse *ANG-2* forward	CTCTGTCTCAGGATGACTCCAG
Mouse *ANG-2* reverse	AGGTGTTGACATCTTTGCAGAAAG
Mouse *EGF* forward	ACTGGTGTGACACCAAGAGGTC
Mouse *EGF* reverse	CCACAGGTGATCCTCAAACACG
Mouse *PIGF* forward	AGTTTCACAGGAGCGTGGCTTG
Mouse *PIGF* reverse	GATCCAGAGTGGCGAGATAACC
Mouse *VEGFA* forward	CTGCTGTAACGATGAAGCCCTG
Mouse *VEGFA* reverse	GCTGTAGGAAGCTCATCTCTCC
Mouse *GAPDH* forward	CATCACTGCCACCCAGAAGACTG
Mouse *GAPDH* reverse	ATGCCAGTGAGCTTCCCGTTCAG
Human *EGF* forward	TGCGATGCCAAGCAGTCTGTGA
Human *EGF* reverse	GCATAGCCCAATCTGAGAACCAC
Human *FGF2* forward	AGCGGCTGTACTGCAAAAACGG
Human *FGF2* reverse	CCTTTGATAGACACAACTCCTCTC
Human *HGF* forward	GAGAGTTGGGTTCTTACTGCACG
Human *HGF* reverse	CTCATCTCCTCTTCCGTGGACA
Human *VEGFA* forward	TTGCCTTGCTGCTCTACCTCCA
Human *VEGFA* reverse	GATGGCAGTAGCTGCGCTGATA
Human *GAPDH* forward	GTCTCCTCTGACTTCAACAGCG
Human *GAPDH* reverse	ACCACCCTGTTGCTGTAGCCAA

### Cell apoptosis, reactive oxygen species, MitoSOX, and mitochondrial permeability transition pore staining

Cells at 60% to 70% confluence were treated with 800M H_2_O_2_ for 24h. A total of 1% FBS was added to avoid severe cell injury, which is optimal to determine the protective effects of the intervention. The cell apoptosis was determined with an AnnexinV-APC/PI apoptosis detection kit (Sony Biotechnology, 3804660) according to the manufacturers instructions. Cell reactive oxygen species (ROS) were determined by the Total Reactive Oxygen Species Assay Kit (Thermo Fisher, 88-5930-74). Mitochondrial superoxide was stained with MitoSOX red indicator (Thermo Fisher, M36008). Mitochondrial permeability transition pore (mPTP) opening was determined with the MitoProbe Transition Pore Assay Kit (Thermo Fisher, M34153).

### Proliferation assay

The effects of MSC-CdM on HUVECs and skin fibroblasts proliferation were determined with the Cell Counting Kit8 assay (Dojindo, CK04). Briefly, 1 10^3^ cells per well (4 replicates per group) were seeded into 96-well plates and cultured in the medium as indicated. At indicated time points, Cell Counting Kit8 solution (10L) and 100L of fresh culture medium were added to each well and incubated at 37C for 1h. The absorbance was observed at 450nm with a microplate reader. The survival/proliferation of cells was calculated as the absorbance of the test wells minus the optical density of the blank wells.

### Tube formation assay

Growth Factor Reduced Matrigel (BD Biosciences, 356231) was plated in 96-well plates and incubated at 37C for 30min. Then, human umbilical vein endothelial cells (HUVECs) were seeded on polymerized Matrigel at 1 10^4^ per well (4 replicates per group), and the medium was added as indicated. A total of 10uM U0126 (MCE, HY-12031) was added in the U0126-treated groups. After incubation at 37C for 4h, tube formation was recorded with an inverted microscope. The total branching points and total tube length were measured with Image-Pro Plus 6 software.

### Protein array

Angiogenic protein concentration in uMSC-CdM and iMSC-CdM was determined with an antibody array (Raybiotec, QAH-ANG-1). This multiplexed sandwich ELISA-based quantitative array platform detects 10 proteins. Pooled conditioned medium from uMSC (*n* = 3) and iMSC (*n* = 3) was used for the experiment according to the manufacturer's instructions. The signals were captured by a laser scanner InnoScan 300 equipped with a Cy3 wavelength and analyzed by the microarray analysis software.

### ATP concentration assessments

A total of 20 10^4^ cells/per well were seeded into 6 well plates, and 2mL DMEM, uMSC-CdM, or iMSC-CdM was added in indicated groups, respectively. The inhibitors including 10uM U0126 (MCE, HY-12031), 20uM SB203580 (MCE, HY-10256), and 10uM LY294002 (MCE, HY-10108) wereadded in indicated groups, respectively. Twenty-four hours later, cells were harvested for in vitro adenosine triphosphate (ATP) concentration analysis. ATP concentrations were determined with an ATP assay kit (Beyotime, S0027) according to the manufacturers instructions.

### Oxygen consumption rate measurement

A Seahorse XFp Analyzer (Agilent Technologies, RRID: SCR_013575) was applied to evaluate the mitochondrial function as previously described [21]. Briefly, 1.5 10^4^ HUVECs were seeded in each well of an XF cell culture microplate with 180L culture medium. U0126 was added at a concentration of 10uM in the iMSC-CdM + U0126 group. Cells were incubated overnight at 37C in 5% CO_2_, and then the culture medium was replaced with 180L of XF Medium. The bioenergetic profile was characterized with the following 4 parameters: (i) basal respiration in assay medium with pyruvate (0.6mM), L-glutamine (6.98mM), and D-glucose (5.78mM); (ii) post inhibition ATP synthase activity by 1.5M oligomycin as well as respiration-driving proton leak and ATP synthesis turnover; (iii) maximal mitochondrial respiratory capacity following treatment with the uncoupling agent 0.5M FCCP (carbonyl cyanide p-trifluoromethoxyphenylhydrazone); and (iv) nonmitochondrial respiration measured posttreatment with 2M complex I inhibitor rotenone and complex III inhibitor antimycin A.

### siRNA transfection

MFN1 siRNA was used to transfect HUVECs using Lipofectamine RNAiMAX (Thermo, 13778-075) according to the manufacturers instructions. HUVECs at 7080% confluence were transfected and incubated for 48h. The transfection efficiency was determined by Western blot analysis.

### MitoTracker staining

The morphology of mitochondria was examined by MitoTracker staining (Cell Signaling, 9074) as previously described [22]. The percentage of mitochondrial fragmentation was calculated by comparing the number of cells with fragmented mitochondria to the total number of cells.

### Western blotting

Protein extracts were separated by sodium dodecyl sulfate-polyacrylamide gel electrophoresis and transferred to polyvinylidene fluoride membranes as previously described [23]. The membranes were incubated with the following antibodies: anti-VEGFA (Proteintech, 66828-1-1g); anti-pERK1/2 (Cell Signaling, 4370), anti-ERK1/2 (Cell Signaling, 4695), anti-MFN1 (Proteintech, 13798-1-AP), anti-MFN2 (Proteintech, 12186-1-AP), anti-pDRP1 ser616 (Cell Signaling, 3455), anti-DRP1 (Proteintech, 12957-1-AP), and anti--actin (Cell Signaling, 3700), at 4C overnight. After washing, the membrane was incubated with the horseradish peroxidase-conjugated secondary antibodies at 37C for 1h. The immunoreactive bands were visualized with enhanced chemiluminescence reagent (Thermo Fisher, 32109) and imaged with the ChemiDoc XRS Plus luminescent image analyzer (Bio-Rad). Densitometric quantification of band intensity from 4 independent experiments was conducted with Image-Pro Plus 6.0 software.

### Statistical analysis

All experiments were performed with at least 3 replicates per group, and the in vitro experiments were repeated at least 3 times. Data are representative of these experiments and are shown as means plus or minus standard error of the mean. The means of multiple groups were compared with the one-way analysis of variance (ANOVA). Statistical analyses wereconducted with GraphPad Prism software, and *P* < .05 was considered statistically significant.

## Results

### Characterization of uMSC and iMSC and preparation of uMSC-CdM/iMSC-CdM

**Fig. 1:**
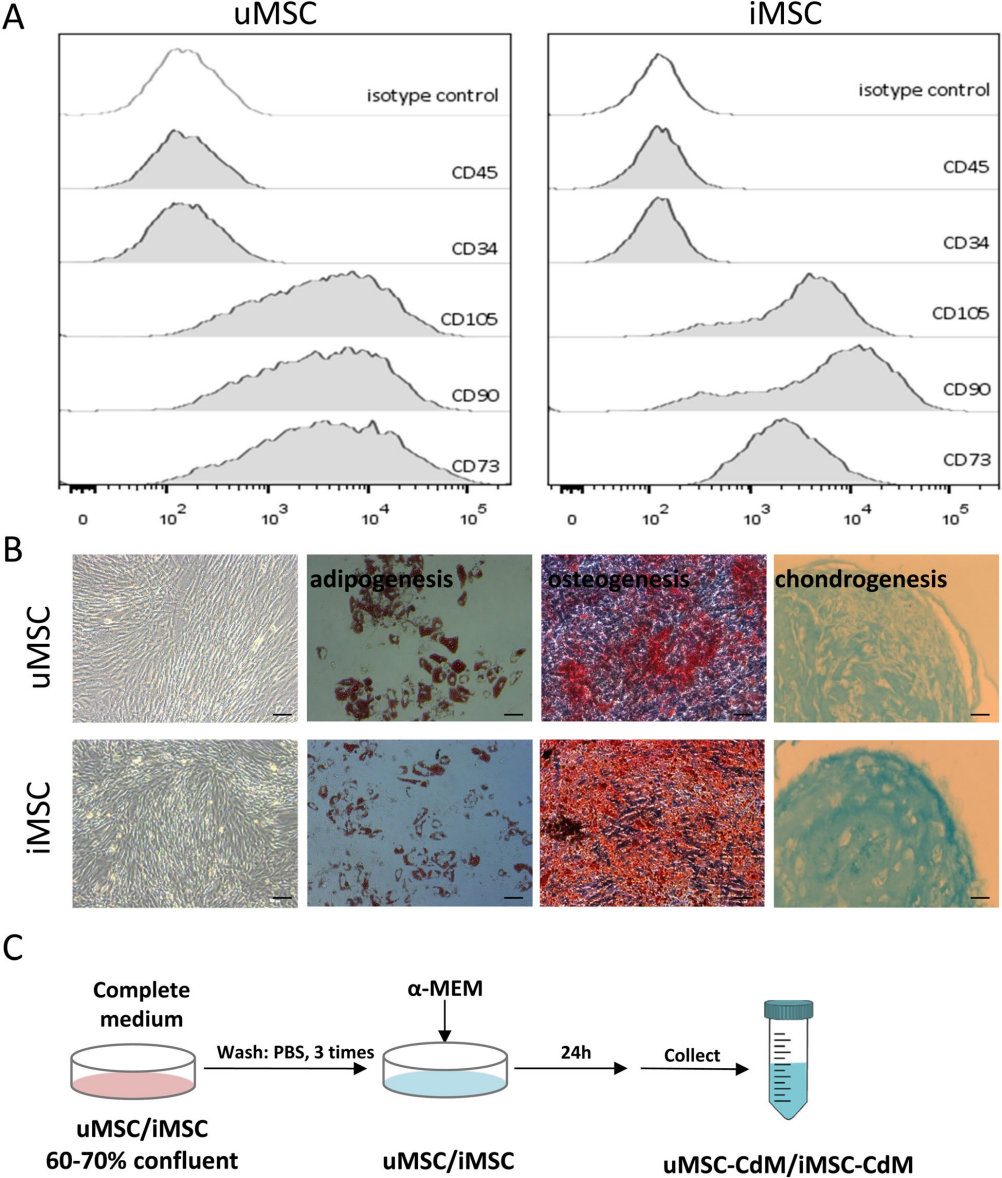
Characterization of mesenchymal stem cells derived from umbilical cord mesenchymal stem cells (uMSCs) and mesenchymal stem cells derived from induced pluripotent stem cells (iMSCs). **a** Surface marker profiling was determined by flow cytometry. **b** Morphology and trilineage differentiation of uMSCs and iMSCs. Scale bar = 100m. **c** Schematic diagram for conditioned medium collection

MSCs at passage 4 were characterized by surface marker profiling and differentiation capacity. Both uMSCs and iMSCs displayed positive expression of MSC markers CD105, CD90 and CD73, and negative expression of CD34 and CD45 (Fig. 1a). The uMSCs and iMSCs showed a spindle shape and multiple differentiation potential toward adipogenesis, chondrogenesis and osteogenesis (Fig. 1b). A schematic diagram of uMSC-CdM and iMSC-CdM preparation is shown in Fig. 1c.

### iMSC-CdM treatment is more effective than uMSC-CdM in accelerating wound closure

**Fig. 2:**
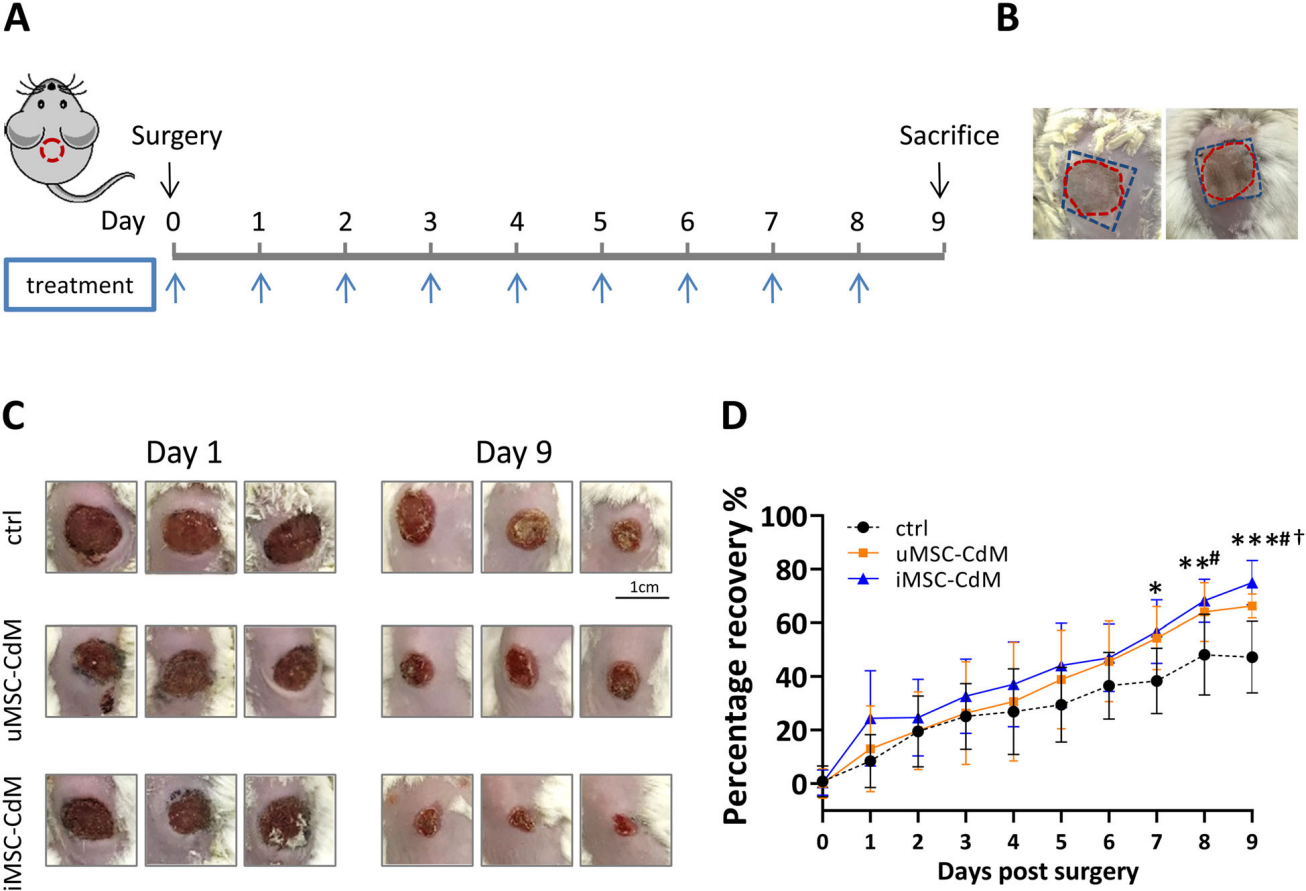
Conditioned medium of iMSCs effectively accelerated cutaneous wound healing. **a** Schematic diagram of animal study. Conditioned medium of mesenchymal stem cells derived from umbilical cord mesenchymal stem cells (uMSC-CdM) and conditioned medium of mesenchymal stem cells derived from induced pluripotent stem cells (iMSC-CdM) was applied daily after the surgery. **b** Schematic diagram depicting delivery method of uMSC-CdM/iMSC-CdM. A sterile cotton pad (blue box) was placed to cover the wound area (red circle) and then uMSC-CdM or iMSC-CdM was topically administered onto the pad. **c** Representative images of the wounds receiving conditioned medium treatment were taken 1 and 9days after surgery. Scale bar = 1cm. **d** The recovery rate was measured daily after treatment. *P < .05 iMSC-CdM vs control group; **P < .01 iMSC-CdM vs control group; ***P < .001 iMSC-CdM vs control group; ^#^*P* < .05 uMSC-CdM vs control group; *P* < .05 iMSC-CdM vs uMSC-CdM group

After wound induction, the wounds were treated daily by topical administration of medium to a sterile cotton pad covering the wound and surrounding area (Fig. 2a, b). Mice in the control group lost weight in the first 4days after wound induction, while mice in the conditioned medium groups maintained their body weight (Supplementary Figure 1). Conditioned medium significantly accelerated wound closure versus the control group (Fig. 2c, d). Notably, the iMSC-CdM treated group showed more prominent therapeutic effects than the uMSC-CdM group (Fig. 2c, d).

### iMSC-CdM treatment inhibits inflammation and increases neovascularization of wounded tissue

**Fig. 3:**
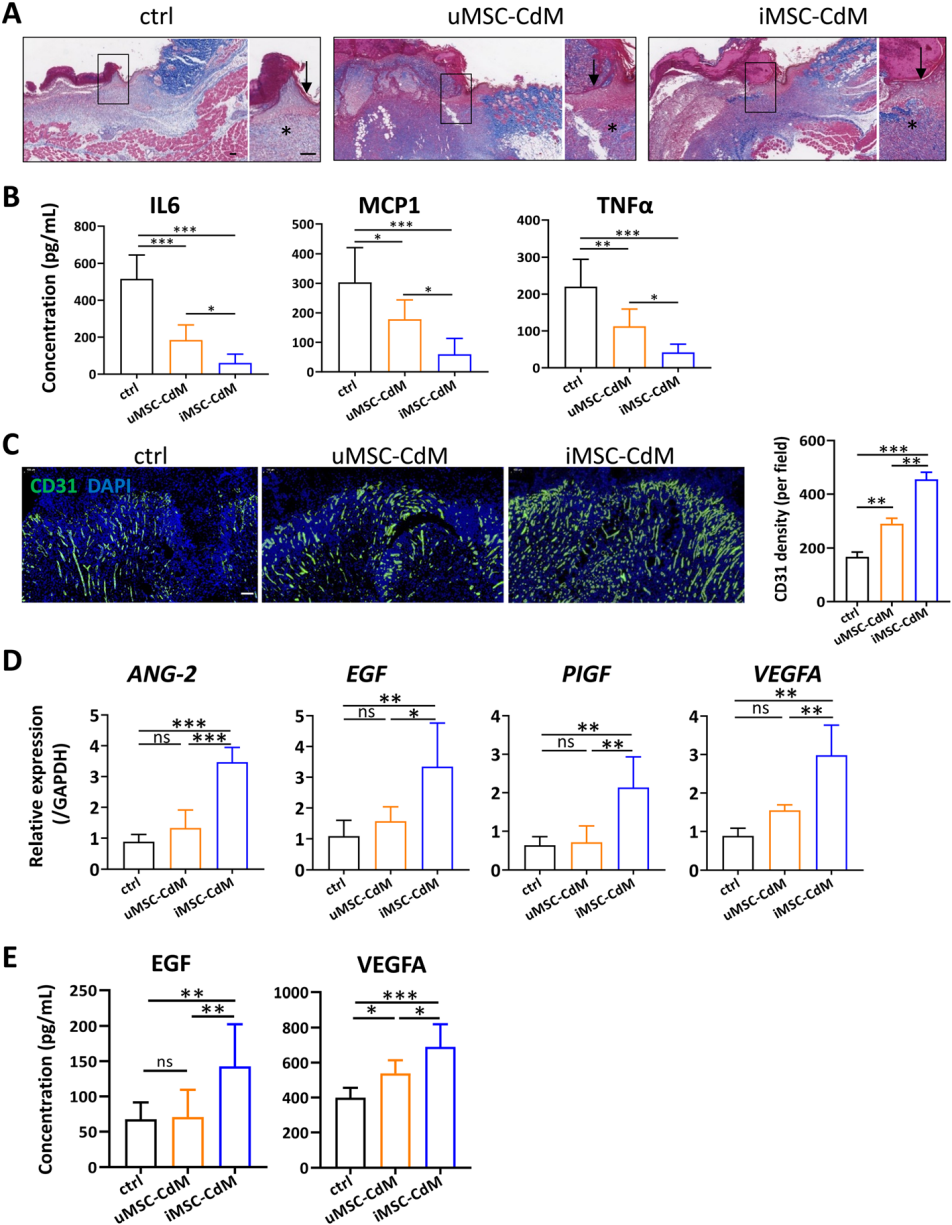
Treatment with iMSC-CdM inhibited inflammation and enhanced angiogenesis. **a** Trichrome staining revealed that the wounds treated with uMSC-CdM and iMSC-CdM had similar granulation tissue at the peri-wound area (arrows indicate keratinocytes; stars indicate granulation tissue). Scale bar = 100m. **b** Inflammatory cytokine expression was determined by a bead-based immunoassay. **c** Representative fluorescence images and quantification of CD31 positive blood vessels. Scale bar = 100m. **d** Expression of pro-angiogenesis factors at the peri-wound area determined by quantitative polymerase chain reaction. **e** EGF and VEGFA protein expression determined by Elisa assay. ns, non-significance, *P < .05, **P < .01, ***P < .001

Trichrome staining of wound sections revealed that uMSC-CdM- and iMSC-CdM-treated mice had similar granulation tissue at 9days after wound induction. By contrast, the control group lacked blue-stained collagen fibers (Fig. 3a). The conditioned medium treatment inhibited inflammatory cytokine secretion, including IL6, MCP1 and TNF, versus the control group, and iMSC-CdM was more effective than uMSC-CdM (Fig. 3b). The other three cytokines, including IFN, IL10 and IL12p70 showed a low expression out of the measuring range limit in all three groups (data not shown). MSCs can activate the angiogenesis, proliferation, migration, and differentiation of the main cell types involved in skin regeneration, including endothelial cells, fibroblasts, and keratinocytes [24]. We isolated skin fibroblasts and determined whether uMSC-CdM/iMSC-CdM affects the proliferation and migration of fibroblasts by CCK8 assay and transwell assay, respectively. As shown in Supplementary Figure 2A, uMSC-CdM and iMSC-CdM increased proliferation of skin fibroblasts at day 3, and there was no difference between the uMSC-CdM group and iMSC-CdM group. The uMSC-CdM and iMSC-CdM did not affect the mobility of skin fibroblasts (Supplementary Figure 2B). Immunostaining showed an enhanced expression of fibroblast marker vimentin in the conditioned medium treated groups compared to the control group, although no significant difference was detected between the uMSC-CdM group and iMSC-CdM group (Supplementary Figure 2C). To evaluate the effects of uMSC-CdM/iMSC-CdM on keratinocytes, immunostaining against keratinocyte marker cytokeratin was performed. The data showed an enhanced cytokeratin expression in the conditioned medium treated groups compared to the control group. Still, there wasno significant difference between the uMSC-CdM treated group and iMSC-CdM treated group (Supplementary Figure 2D). Collectively, these data suggested uMSC-CdM and iMSC-CdM have positive effects on keratinocytes and fibroblasts. However, there was no significant difference between the uMSC-CdM and iMSC-CdM groups. Angiogenesis is an essential component of wound repair, as vessels support cells at the wound site with nutrition and oxygen. We then determined the capillary density of the peri-wound area by immunostaining with anti-CD31 (a marker of endothelial cells). Capillary density was enhanced in the conditioned medium treated groups versus the control group, with more significant enhancement in the iMSC-CdM group than in the uMSC-CdM group (Fig. 3c). The expression level of angiogenesis factors, including *angiopoietin 2 (ANG-2)*, *EGF, placenta growth factor (PIGF)* and *VEGFA*, at the peri-wound area, were further measured by quantitative polymerase chain reaction analysis. While the uMSC-CdM treatment showed marginally higher expressions of *ANG-2*, *EGF*, *PIGF* and *VEGFA* versus the control (statistically not significant), the iMSC-CdM treatment increased the expression of the cytokines as mentioned above (Fig. 3d). Further, we measured the protein expression of EGF and VEGFA by ELISA assay. There was no significant difference in EGF protein expression between the control and uMSC-CdM treated group (Fig. 3e). Nevertheless, the VEGFA expression was significantly higher in the conditioned medium treated groups compared to the control group. Notably, the iMSC-CdM-treated group exhibited a higher EGF and VEGF expression compared to the control and uMSC-CdM treated groups, respectively (Fig. 3e). Taken together, these data showed that external application of iMSC-CdM inhibited inflammation and increased neovascularization of wounded tissue.

### The cytoprotective effects and angiogenic potential of iMSC-CdM on HUVECs

**Fig. 4:**
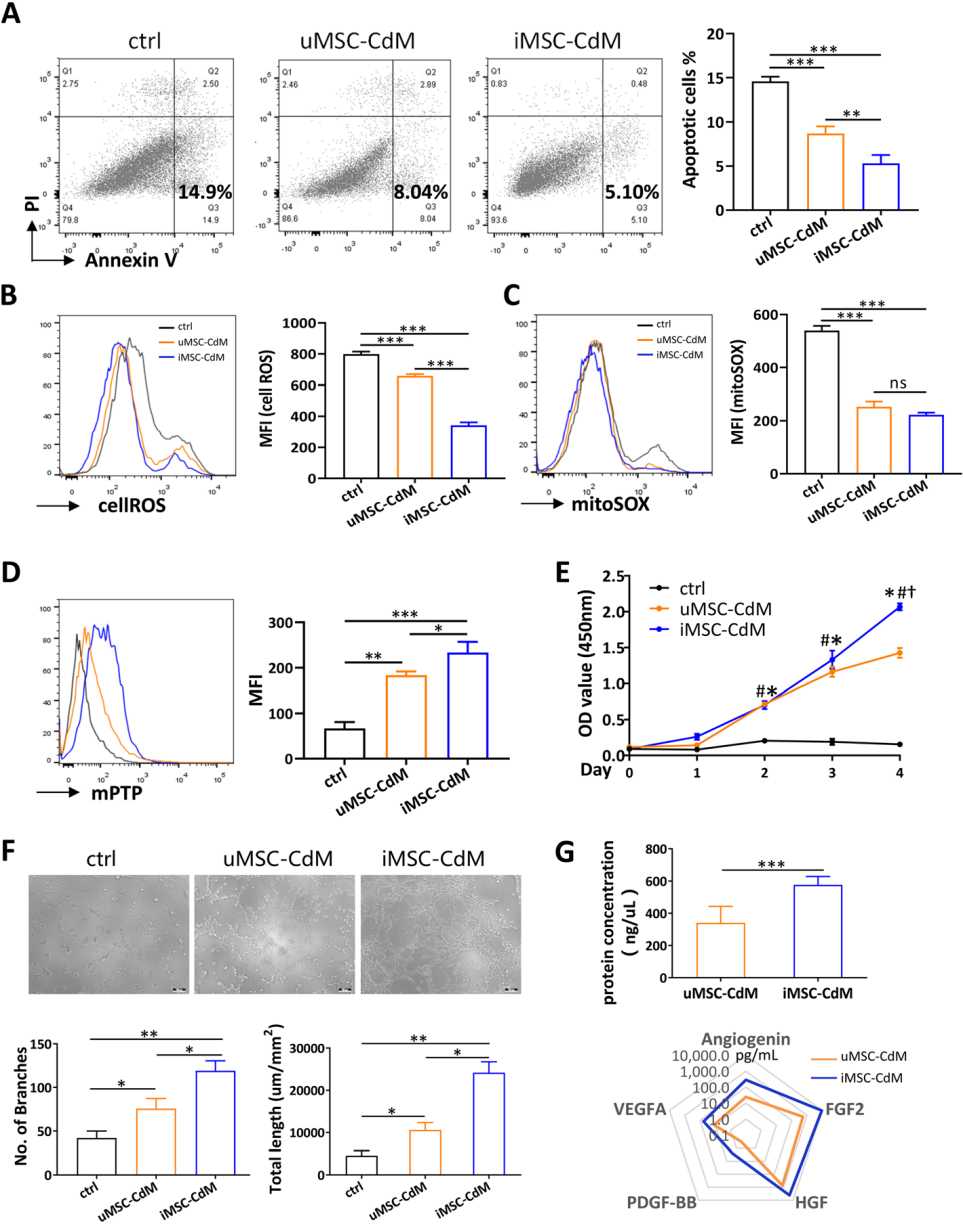
The cytoprotective effects of iMSC-CdM on H_2_O_2_ treated HUVECs. After H_2_O_2_ treatment, **a** apoptosis, **b** cell reactive oxygen species, **c** mitochondrial reactive oxygen species, and **d** mitochondrial permeability transition pore (mPTP) opening were determined by flow cytometry. MFI, mean fluorescent intensity. ns, non-significance, *P < .05, **P < .01, ***P < .001. **e** Cell proliferation was determined by Cell Counting Kit8 assay. *P < .05 iMSC-CdM vs control group; #*P* < .05 uMSC-CdM vs control group; *P* < .05 iMSC-CdM vs uMSC-CdM group. **f** Representative light photomicrographs and quantitative analysis of HUVEC tube formation assay after uMSC-CdM/iMSC-CdM treatment. Scale bar = 100m. *P < .05, **P < .01. **g** Protein concentration and expression levels of angiogenic factors in uMSC-CdM and iMSC-CdM. ***P < .001

Enhanced angiogenesis in vivo suggested a protective function of conditioned medium on endothelial cells. We thus determined the cytoprotective effects of conditioned medium on HUVECs under H_2_O_2_-induced stress. H_2_O_2_ induced HUVEC apoptosis in controls, and conditioned medium treatment significantly reduced apoptosis (Fig. 4a). The iMSC-CdM group showed a more protective effect than the uMSC-CdM group (Fig. 4a). Similarly, conditioned medium significantly reduced levels of cellular ROS induced by H_2_O_2_ treatment, and iMSC-CdM was more effective in reducing cellular ROS than uMSC-CdM (Fig. 4b). We further determined the levels of mitochondrial ROS and showed that iMSC-CdM and uMSC-CdM were equivalent in inhibiting mitochondrial ROS (Fig. 4c). The mitochondrial permeability transition pore (mPTP) opening was analyzed as the capacity of mitochondria to retain the dye calcein-AM in presence of 200M CaCl_2_. The data showed that H_2_O_2_ markedly induced mPTP opening, as evidenced by reduced calcein-AM intensity (Fig. 4d). The effect of H_2_O_2_-induced mPTP opening was prevented by the conditioned medium treatment, and iMSC-CdM provided more protection than uMSC-CdM, as evidenced by increased calcein-AM intensity (Fig. 4d). Under normal culture conditions, conditioned medium treatment significantly increased proliferation rate in HUVECs, and at day 4, iMSC-CdM had a more substantial effect than uMSC-CdM (Fig. 4e). The angiogenic capacity of the conditioned medium was analyzed by tube formation assay, which showed that iMSC-CdM treatment increased endothelial network formation more than uMSC-CdM treatment (Fig. 4f). With equivalent cell numbers, the protein concentration of iMSC-CdM was higher than uMSC-CdM, and the levels of angiogenic factor angiogenin, VEGFA, PDGF-BB, HGF and FGF2 were higher in iMSC-CdM than they were in uMSC-CdM (Fig. 4g). Collectively, these data suggested that iMSC-CdM presented more significant cytoprotective effects and angiogenic potential than uMSC-CdM.

### iMSC-CdM enhanced energy metabolism and angiogenesis via activating ERK signaling pathway

**Fig. 5:**
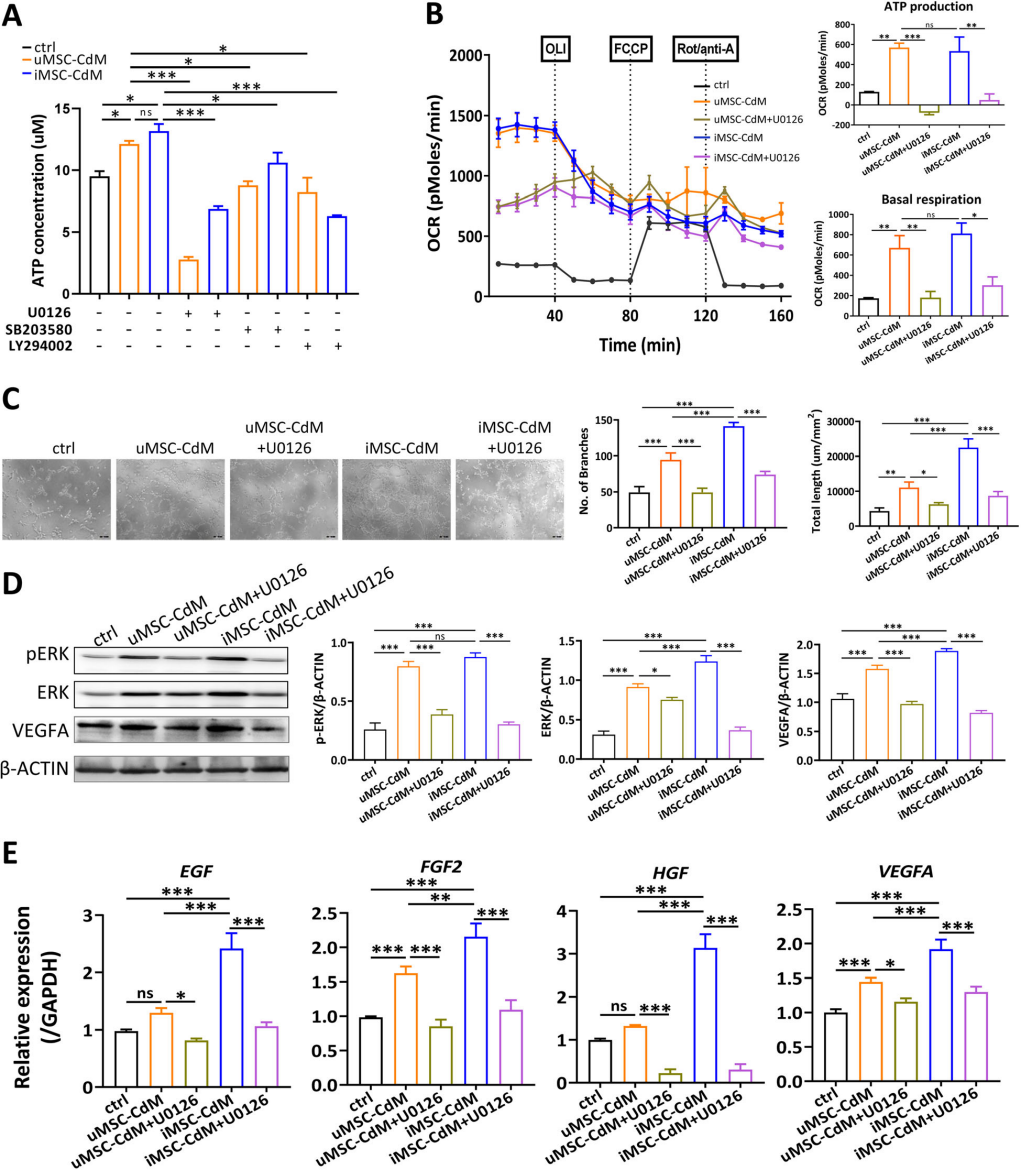
iMSC-CdM enhanced energy metabolism and angiogenesis via the ERK pathway. **a** ATP concentrations were determined in conditioned medium-treated HUVECs with pathway inhibitors as indicated. **b** Respiratory potential of conditioned medium-treated HUVECs quantified with the Seahorse Metabolic Analyzer. Real-time measurements of oxygen consumption rate (OCR) were obtained basally and then after treatment with oligomycin (ATP synthase inhibitor), carbonyl cyanide p-trifluoromethoxyphenylhydrazone (FCCP, electron transport chain accelerator), and rotenone plus antimycin A (electron transport chain inhibitors). **c** Representative photomicrographs and quantitative analysis of tube formation assay in conditioned medium-treated HUVECs with and without ERK inhibitor U0126. Scale bar = 100m. **d** Representative immunoblot images and quantitative analysis in conditioned medium-treated HUVECs with and without ERK inhibitor U0126. **e** Expression of pro-angiogenesis factors in conditioned medium-treated HUVECs with and without ERK inhibitor U0126. ns, non-significance, *P < .05, **P < .01, ***P < .001

To evaluate the molecular mechanisms by which conditioned medium facilitated angiogenesis, HUVECs were treated with pathway inhibitors U0126 (ERK inhibitor), SB203580 (p38 MAPK inhibitor), and LY294002 (PI3K/Akt inhibitor), and ATP concentrations were measured. Among the 3 inhibitors, U0126 showed the most prominent inhibition in ATP content in both the uMSC-CdM and iMSC-CdM groups, suggesting that the ERK signaling pathway might play a predominant role in regulating conditioned medium-mediated cytoprotection (Fig. 5a). The effects of conditioned medium on the energy metabolism of HUVECs were then examined. The oxygen consumption rate (OCR) of HUVECs was determined with an extracellular flux analyzer. Conditioned medium treatment significantly increased the basal respiratory capacity and ATP production of HUVECs (Fig. 5b). Supplementation of U0126 significantly decreased basal respiratory capacity and ATP production mediated by conditioned medium treatment (Fig. 5b). Similarly, iMSC-CdM treatment enhanced tube formation (Fig. 5c), increased protein expression of ERK, p-ERK and VEGFA (Fig. 5d), and induced angiogenic factor expression including *EGF, FGF2, HGF* and *VEGFA* (Fig. 5e) more than uMSC treatment did. U0126 supplementation partially neutralized the effects of iMSC-CdM on tube formation (Fig. 5c), protein expression (Fig. 5d), and angiogenic cytokine expression (Fig. 5e). Collectively, these data suggested that iMSC-CdM enhanced energy metabolism and angiogenesis by activating the ERK signaling pathway.

### iMSC-CdM inhibited H_2_O_2_-induced mitochondrial fragmentation and apoptosis of HUVECs

**Fig. 6:**
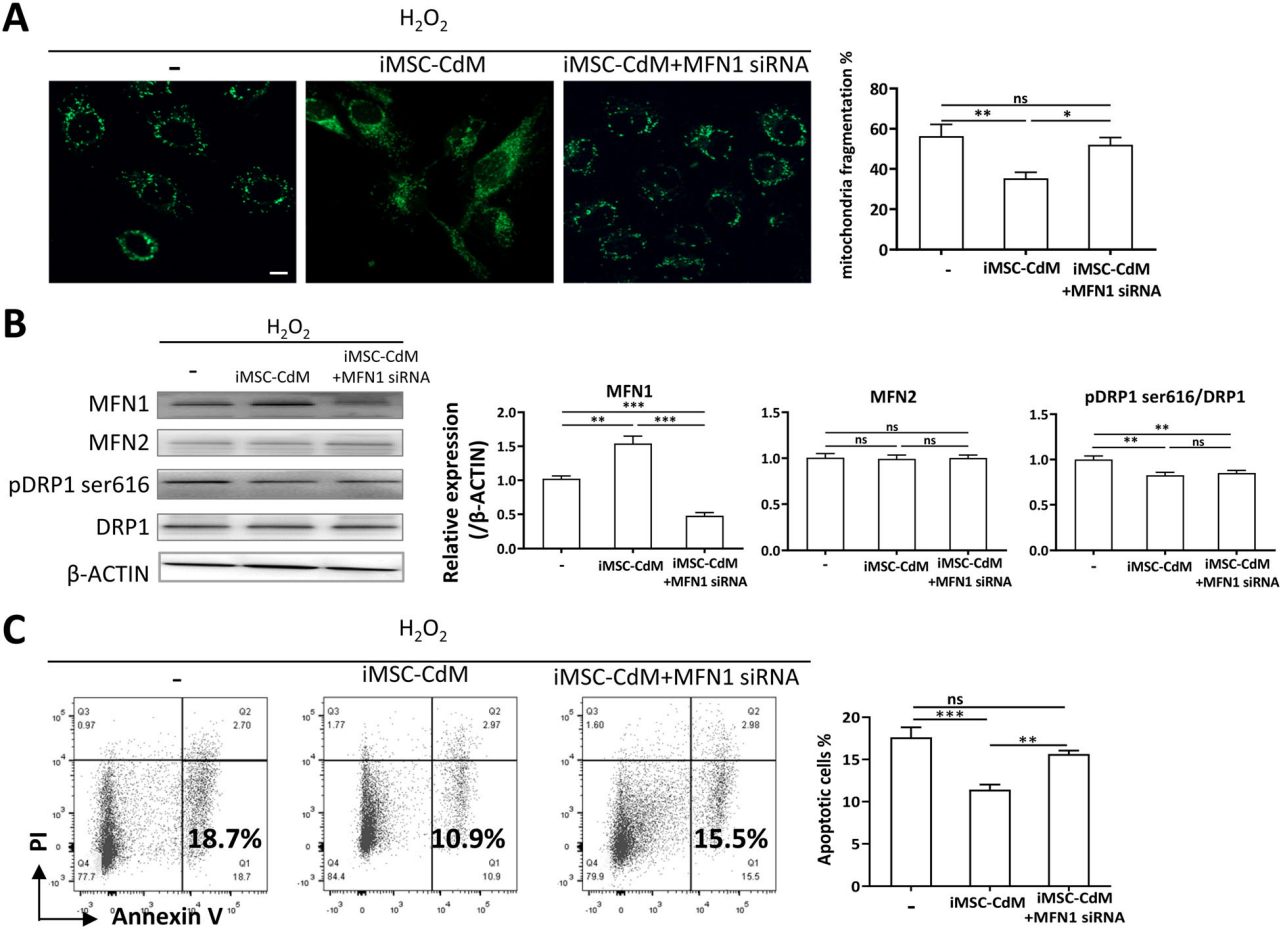
iMSC-CdM treatment suppresses H_2_O_2_-induced mitochondria fragmentation and apoptosis of HUVECs. **a** Representative images and quantitative analysis of fragmented mitochondria in H_2_O_2_-treated HUVECs. Scale bar = 50 um. **b** Western blotting and quantitative analysis for the expression of MFN1, MFN2, DRP1 and pDRP1 ser616 in H_2_O_2_-treated HUVECs. **c** Apoptosis was determined by flow cytometry. ns, non-significance, *P < .05, **P < .01, ***P < .001

Mitochondria are the power generators of the cell, converting oxygen and nutrients into ATP. High ROS levels may release a ROS burst leading to the destruction of mitochondria or even the cell. We have shown iMSC-CdM protected HUVECs from H_2_O_2_ induced-apoptosis and ROS production (Fig. 4), and iMSC-CdM increased the basal respiratory capacity and ATP production of HUVECs (Fig. 5a, b). We then determined whether iMSC-CdM regulated H_2_O_2_-induced mitochondrial fragmentation in HUVECs. We showed the iMSC-CdM treatment significantly reduced H_2_O_2_-induced mitochondrial fragmentation in HUVECs (Fig. 6a). Western blotting demonstrated downregulation of p-DRP1 ser616 and upregulation of MFN1 in the iMSC-CdM treated group compared to the non-treated group (Fig. 6b). There was no difference in MFN2 expression between the iMSC-CdM treated and non-treated groups (Fig. 6b). Moreover, the iMSC-CdM treatment decreased H_2_O_2_-induced apoptosis compared to the non-treated group (Fig. 6c). We then determined whether iMSC-CdM exerted protective effects through regulating mitochondria dynamics. As MFN1 was upregulated in the iMSC-CdM treated group compared to the non-treated group (Fig. 6b), we applied MFN1-siRNA to suppress its expression in the iMSC-CdM treated group, and then mitochondria fragmentation and apoptosis were examined. The elevation of MFN1 expression induced by iMSC-CdM treatment under H_2_O_2_ stimulation was inhibited by MFN1 siRNA (Fig. 6b). Notably, the MFN1 siRNA treatment partially neutralized the effects of iMSC-CdM on mitochondria fragmentation (Fig. 6a) and apoptosis (Fig. 6c). A mitochondrial energy metabolism PCR array was applied to determine the expression profile of 84 key genes related to mitochondria biogenesis and function. The result showed 76/84 (90.5%) genes were downregulated in the iMSC-CdM + MFN1 siRNA group compared to the iMSC-CdM group (Supplementary Figure 3), including genes encoding subunits of ATP synthesis, complex I, complex II, complex III (Table 2). Collectively, these observations indicated that iMSC-CdM protected cells from H_2_O_2_-induced injury by regulating mitochondria dynamics.

**Table 2:** List of the genes up- or downregulated (>2 fold) in the iMSC-CdM + MFN1 siRNA group with respect to iMSC-CdM group

Position	Symbol	RefSeq number	Description	Fold regulation
A01	*ATP5A1*	NM_004046	ATP synthase, H+ transporting, mitochondrial F1 complex, alpha subunit 1, cardiac muscle	2.33
A03	*ATP5C1*	NM_005174	ATP synthase, H+ transporting, mitochondrial F1 complex, gamma polypeptide 1	2.21
A04	*ATP5F1*	NM_001688	ATP synthase, H+ transporting, mitochondrial Fo complex, subunit B1	2.29
A05	*ATP5G1*	NM_005175	ATP synthase, H+ transporting, mitochondrial Fo complex, subunit C1 (subunit 9)	2.44
A08	*ATP5H*	NM_006356	ATP synthase, H+ transporting, mitochondrial Fo complex, subunit d	2.03
A11	*ATP5J2*	NM_004889	ATP synthase, H+ transporting, mitochondrial Fo complex, subunit F2	2.71
A12	*ATP5L*	NM_006476	ATP synthase, H+ transporting, mitochondrial Fo complex, subunit G	3.01
B02	*COX4I1*	NM_001861	Cytochrome c oxidase subunit IV isoform 1	2.22
B04	*COX5B*	NM_001862	Cytochrome c oxidase subunit Vb	2.03
B06	*COX6A2*	NM_005205	Cytochrome c oxidase subunit VIa polypeptide 2	2.32
B08	*COX6C*	NM_004374	Cytochrome c oxidase subunit Vic	2.3
C02	*NDUFA1*	NM_004541	NADH dehydrogenase (ubiquinone) 1 alpha subcomplex, 1, 7.5kDa	2.09
C03	*NDUFA10*	NM_004544	NADH dehydrogenase (ubiquinone) 1 alpha subcomplex, 10, 42kDa	2.09
C04	*NDUFA11*	NM_175614	NADH dehydrogenase (ubiquinone) 1 alpha subcomplex, 11, 14.7kDa	2.14
C06	*NDUFA3*	NM_004542	NADH dehydrogenase (ubiquinone) 1 alpha subcomplex, 3, 9kDa	2.89
C09	*NDUFA6*	NM_002490	NADH dehydrogenase (ubiquinone) 1 alpha subcomplex, 6, 14kDa	3.77
C10	*NDUFA8*	NM_014222	NADH dehydrogenase (ubiquinone) 1 alpha subcomplex, 8, 19kDa	2.57
C11	*NDUFAB1*	NM_005003	NADH dehydrogenase (ubiquinone) 1, alpha/beta subcomplex, 1, 8kDa	3.36
C12	*NDUFB10*	NM_004548	NADH dehydrogenase (ubiquinone) 1 beta subcomplex, 10, 22kDa	2.52
D05	*NDUFB6*	NM_182739	NADH dehydrogenase (ubiquinone) 1 beta subcomplex, 6, 17kDa	2.46
D06	*NDUFB7*	NM_004146	NADH dehydrogenase (ubiquinone) 1 beta subcomplex, 7, 18kDa	2.66
D07	*NDUFB8*	NM_005004	NADH dehydrogenase (ubiquinone) 1 beta subcomplex, 8, 19kDa	2.5
D08	*NDUFB9*	NM_005005	NADH dehydrogenase (ubiquinone) 1 beta subcomplex, 9, 22kDa	2.02
D09	*NDUFC1*	NM_002494	NADH dehydrogenase (ubiquinone) 1, subcomplex unknown, 1, 6kDa	2.48
D10	*NDUFC2*	NM_004549	NADH dehydrogenase (ubiquinone) 1, subcomplex unknown, 2, 14.5kDa	24.21
D12	*NDUFS2*	NM_004550	NADH dehydrogenase (ubiquinone) Fe-S protein 2, 49kDa (NADH-coenzyme Q reductase)	2.02
E02	*NDUFS4*	NM_002495	NADH dehydrogenase (ubiquinone) Fe-S protein 4, 18kDa (NADH-coenzyme Q reductase)	3.19
E04	*NDUFS6*	NM_004553	NADH dehydrogenase (ubiquinone) Fe-S protein 6, 13kDa (NADH-coenzyme Q reductase)	2.73
E06	*NDUFS8*	NM_002496	NADH dehydrogenase (ubiquinone) Fe-S protein 8, 23kDa (NADH-coenzyme Q reductase)	2.36
E07	*NDUFV1*	NM_007103	NADH dehydrogenase (ubiquinone) flavoprotein 1, 51kDa	2.46
F01	*SDHC*	NM_003001	Succinate dehydrogenase complex, subunit C, integral membrane protein, 15kDa	4.59
F03	*UQCR11*	NM_006830	Ubiquinol-cytochrome c reductase, complex III subunit XI	4.87
F08	*UQCRQ*	NM_014402	Ubiquinol-cytochrome c reductase, complex III subunit VII, 9.5kDa	2.77
F09	*ARRDC3*	NM_020801	Arrestin domain containing 3	3.08
F10	*ASB1*	NM_001040445	Ankyrin repeat and SOCS box containing 1	6.03
F11	*CYB561D1*	NM_182580	Cytochrome b-561 domain containing 1	11.48
F12	*DNAJB*	NM_006145	DnaJ (Hsp40) homolog, subfamily B, member 1	2.15
G01	*EDN1*	NM_001955	Endothelin 1	2.14
G04	*HSPA1B*	NM_005346	Heat shock 70kDa protein 1B	2.34
G06	*MitoH1*	r4_NC_012920	Polycistronic_H1_3	2.95

Changes in gene expression between groups were evaluated using RT2 Profiler 96-well PCR array plates. Data analysis was done by the 2^CT^ method on the manufacturers web portal (https://geneglobe.qiagen.com/cn/analyze), (QIAGEN, CA, USA)

## Discussion

MSCs have been considered a promising source for cutaneous wound healing. However, accumulating studies showed that MSCs functioned mainly through immune regulation and paracrine effects rather than direct differentiation [25, 26]. Based on the principle of the paracrine mechanism, MSC conditioned medium contains the secretome of the MSCs and is, therefore, a rich source of paracrine factors. MSCs from different sources have similar phenotypic characteristics but differ in secretome profile [27, 28]. In the current study, we compared the therapeutic effects of iMSC-CdM and uMSC-CdM in treating cutaneous wound healing, and several major findings are as follows: first, iMSC-CdM accelerated wound closure more than uMSC-CdM did (Fig. 2). Histological examinations showed an increase in endothelial marker CD31 and angiogenic factors expression in the peri-wound area in the iMSC-CdM group compared to the uMSC-CdM group (Fig. 3), suggesting the enhanced angiogenesis might involve in iMSC-CdM mediated therapeutics. Following these findings, in vitro studies revealed that iMSC-CdM induced HUVEC proliferation, tube formation, and angiogenic factor expression through activating ERK pathway (Fig. 4eg; Fig. 5ce). The excessive reactive oxygen species (ROS) accumulated in the wound is an essential factor in delayed healing. Long-term instability and high concentrations of ROS will eventually lead to insufficient neovascularization, making blood supply and nutritional requirements unable to meet the needs of wound healing [29]. Since we observed enhanced angiogenesis in the iMSC-CdM treated group in vivo, we examined whether iMSC-CdM exerted a protective role in oxidative stress-induced cell injury in vitro. H_2_O_2_ was applied as the inducer of ROS. The result demonstrated that iMSC-CdM prevented HUVECs from apoptosis, reduced cellular ROS and mitochondrial ROS levels induced by H_2_O_2_ (Fig. 4ac). The H_2_O_2_ induced mPTP openings may release a ROS burst leading to the destruction of mitochondria or even the cell. The iMSC-CdM treatment prevented HUVECs from H_2_O_2_ induced mPTP opening (Fig. 4d). Mitochondria are an important source of ROS generation and energy production. In Fig. 5a, b, we measured the effects of iMSC-CdM on HUVECsenergy metabolism and showed iMSC-CdM increased the basal respiratory capacity and ATP production. Collectively, these data suggested the role of iMSC-CdM on mitochondria function. As highly dynamic organelles, mitochondria undergo coordinated cycles of fission and fusion, referred to as mitochondrial dynamics, to maintain their shape, distribution, and size [30]. We thus determined whether iMSC-CdM regulated mitochondrial dynamics in HUVECs. The data showed iMSC-CdM reduced H_2_O_2_-induced mitochondrial fragmentation and apoptosis, accompanied by decreased pDRP1-ser616 and increased MFN1 expression. These data revealed that iMSC-CdM mediated cytoprotective effects by regulating mitochondria dynamics. In summary, the in vivo data suggested iMSC-CdM accelerated wound healing via enhanced angiogenesis, the in vitro data supported the role of iMSC-CdM in pro-angiogenesis, and the regulation of mitochondria dynamic balance might play a role in iMSC-CdM mediated cytoprotection under oxidative stress.

MSC-based therapy is promising in tissue regeneration and wound repair because it can enhance re-epithelialization, increase angiogenesis, modulate inflammation, and regulate extracellular matrix remodeling [31]. In previous studies on wound healing, MSCs have been injected locally into or around the wound area. However, the diseased microenvironment is not conducive to MSC survival and retention, which limits treatment effects. Alternatively, applying the MSC-conditioned mediumwhich contains the paracrine factorsdirectly to the wound has considerable therapeutic value [32, 33]. Hu et al. reported that injections of conditioned medium from bone-marrow-derived MSCs attenuated hypertrophic scar formation and accelerated re-epithelization in a rabbit ear wound model [34]. Sun et al. showed that Whartons jelly-derived MSC-CdM significantly accelerated wound closure and enhanced wound healing quality versus a positive control (epidermal growth factor) [12]. However, MSCs derived from different sources have different secretory profiles, which might affect their potential therapeutic values. More research is needed to determine the optimal source of MSCs for wound treatment.

iMSCs are emerging as a new source of MSCs due to their potential to overcome several limitations of adult MSCs. First, during in vitro expansion, MSCs derived from adult tissues gradually acquire a senescent phenotype, while iMSCs display greater expansion capacity with differentiation potential and normal karyotypes for over 120 doublings [6]. Second, donor age is a decisive factor for the length of the cultivation lifespan and quality of adult MSCs, which hobbles the application of MSCs isolated from aged donors [4]. Alternatively, irrespective of donor age or cell type, iMSCs acquire a rejuvenation-associated gene signature during the reprogramming process [35]. The telomerase activity of iMSCs is 10 times greater than that of adult bone marrow MSCs [6]. Third, adult MSCs are complex cell populations that exhibit intraheterogeneity, which might be one of the explanations for the inconsistent clinical efficacy. iMSCs may represent a more homogenous population as they grew from a single induced pluripotent stem cell clone. More importantly, studies have highlighted the superiority of iMSCs over the other sources of MSCs for the same applications. Lian et al. demonstrated that iMSCs functioned better than native bone marrow MSCs to recover blood perfusion in a mouse hindlimb ischemia model [6]. Liang et al. reported that iMSCs were more effective than bone marrow MSCs in restoring cigarette smoke-induced cardiac dysfunction via inhibiting the NF-kB pathway [9]. iMSC-CdM reduced cigarette smoke medium-induced mitochondrial ROS in airway smooth muscle cells [10]. Soontararak et al. reported that iMSCs were equivalent to, or in some cases, superior to conventional adipose-derived MSCs in treating inflammatory bowel disease in terms of alleviating clinical signs of colitis and stimulating intestinal healing [36]. These data point to iMSCs as a sustainable source of MSCs for clinical applications. In line with these results, our data showed that iMSC-CdM was superior to uMSC-CdM to treat wounds. With the same seeded cell number, iMSC-CdM yielded a higher protein concentration, which contained a higher level of angiogenesis factors, including angiogenin, VEGFA, PDGF-BB, HGF and FGF2, than uMSC-CdM. At the cellular level, iMSC-CdM induced HUVEC proliferation and tube formation, increased cellular ATP levels and energy metabolism more than uMSC-CdM did. At the organism level, iMSC-CdM treatment significantly reduced inflammatory cytokine expression and enhanced angiogenesis at the peri-wound area. Excessive production of ROS causes oxidative damage, which is the leading cause of non-healing chronic wounds [37]. We measured the effects of conditioned medium on HUVECs under H_2_O_2_ stress. The protective effects of iMSC-CdM on HUVECs were more evident than uMSC-CdM in H_2_O_2_ induced apoptosis. Excessive or inappropriately localized ROS induced cell death, apoptosis and senescence [38]. The main sources of cellular ROS are mitochondria and NADPH oxidases [39]. Cellular ROS and mitochondrial ROS were measured in H_2_O_2_-treated HUVECs. iMSC-CdM showed superior capacity in reducing cellular ROS and equivalent capacity in reducing mitochondrial ROS compared to uMSC-CdM. The mPTP plays a crucial physiological role in maintaining healthy mitochondrial homeostasis. At higher ROS levels, longer mPTP openings may release excessive ROS, leading to the destruction of mitochondria or even the cell [40]. The data showed that iMSC-CdM prevented H_2_O_2_-induced mPTP opening more effectively than uMSC-CdM did. Mechanistically, iMSC-CdM induced angiogenesis and enhanced energy metabolism mainly through activating the ERK pathway. The ERK inhibitor U0126 partially neutralized the effects of iMSC-CdM on HUVECs, including the ATP levels, energy metabolism and expression of angiogenesis factors.

Mitochondria serve as the energy factory of cells; they maintain cellular homeostasis by participating in ATP production, calcium homeostasis, oxidative stress response, and apoptosis [41]. Mitochondria are constantly moving and undergoing morphologic changes controlled by the mitochondrial fusion and fission process. Mitochondria fusion, regulated by MFN1, MFN2, and OPA1 mitochondrial dynamin-like GTPase (OPA1), generates mitochondrial tubule networks. Mitochondria fission, regulated by DRP1 and fission-1 (FIS1), forms smaller individual mitochondria [42]. The imbalance between fusion and fission contributes to endothelial dysfunction [43]. Under pathological conditions, altered mitochondrial dynamics, characterized by increased fission and decreased fusion, lead to accumulation of small dysfunctional mitochondria, loss of mitochondrial networks and increased mitochondrial ROS [43, 44]. DRP1 knockdown attenuates cellular ROS and mitochondria ROS production in high glucose-treated HUVECs [45]. Our data showed HUVECs exhibited a small and round mitochondrion under H_2_O_2_ stimulation. The iMSC-CdM treatment mitigated mitochondria fragmentation and apoptosis in H_2_O_2_ treated HUVECs, accompanied by decreased pDRP1-ser616 and increased MFN1 expression, and these effects were abolished in part by MFN1-siRNA, suggesting iMSC-CdM mediated cytoprotective effects by regulating mitochondria dynamics.

In the current study, the conditioned medium was topically administered to a cotton pad that covered the wound area. In a preliminary investigation, the conditioned medium was injected around the wound area every 3days after the injury. Unfortunately, this treatment was not beneficial and even seemed to worsen the healing process. Excessive inflammation caused by repeated injection might be one explanation for the observed effects- increased inflammatory cytokine IFN was detected in the peripheral lymph nodes, and excessive immune cell infiltration was found in the peri-wound area (data not shown). In light of the beneficial effects of the conditioned medium in vitro, we modified the delivery method according to previously published protocols [12]. In Sun et al.s study, MSC-conditioned medium-hydrogel was pipetted onto a radiation wound in rats every 2days [12]. Here, the protocol was modified by masking the wound with a cotton pad and then pipetting conditioned medium onto the pad. This method allowed well-proportioned distribution of the conditioned medium and made it possible to show that conditioned medium was beneficial in accelerating wound healing. This highlights the importance of the delivery method in MSC-based cell-free therapy in treating various diseases. However, the detailed mechanisms of the healing process warrant further investigation.

This study has limitations. Firstly, we showed the protein concentration of iMSC-CdM was much higher than uMSC-CdM based on equivalent cell numbers. The higher concentration of pro-angiogenic factors angiogenin, VEGFA, FGF2, PDGF-BB, and HGF in iMSC-CdM might partially explain the superior pro-angiogenesis effects as compared to uMSC-CdM. These data suggested iMSC had a more active secretion function compared to uMSC. However, the comparison of the contents in iMSC-CdM and uMSC-CdM, as well as the role of the critical components in iMSC-CdM mediated skin regeneration, needs to be determined by high-performance liquid chromatography-electrospray tandem mass spectrometry (HPLC-MS) and gain and loss-of-function approaches in future studies. Secondly, the current study supports a pro-angiogenic role of iMSC-CdM on endothelial cells during wound healing. The effects of iMSC-CdM on fibroblasts and keratinocytes and whether they contribute to the healing process need further investigation. Third, excessive production of ROS or impaired ROS detoxification causes oxidative damage, which is the leading cause of non-healing chronic wounds. The H_2_O_2_-induced ROS overproduction was applied to establish an oxidative stress-induced cell injury in the current study. However, the in vitro model using H_2_O_2_ only partially mimicked the diseased microenvironment at a certain stage. Application of other stressors, such as hypoxia, can be helpful for a more comprehensive understanding of how iMSC-CdM affects the wound healing process at different stages.

## Conclusion

Collectively, the data showed that iMSC-CdM has more significant therapeutic potential than uMSC-CdM in treating cutaneous skin injury in mice. As a novel cell-free therapeutic approach, iMSC-CdM avoids the ethical issues of cell transplantation and the risk of tumorigenesis. These data support the use of iMSC-CdM for enhancing wound healing or other tissue regeneration.

## Supplementary Information

**Additional file 1.**

## Data Availability

The data that support the findings of this study are available on request from the corresponding author.

## Abbreviations

MSC: Mesenchymal stem cell
uMSC: Umbilical cord MSCs
uMSC-CdM: Conditioned medium from uMSCs
iMSC: Induced pluripotent stem cells derived MSCs
iMSC-CdM: Conditioned medium from iMSCs
HUVEC: Human umbilical vein endothelial cell
CD: Cluster of differentiation
EGF: Epidermal growth factor
ANG-2: Angiopoietin 2
PIGF: Placenta growth factor
VEGFA: Vascular endothelial growth factor A
HGF: Hepatocyte growth factor
FGF2: Fibroblast growth factor 2
IL6: Interleukin 6
MCP1: Monocyte chemoattractant protein-1
TNF: Tumor necrosis factor-alpha
IFN: Interferon
IL10: Interleukin 10
IL12p70: Interleukin 12p70
GAPDH: Glyceraldehyde 3-phosphate dehydrogenase
ROS: Reactive oxygen species
mPTP: Mitochondrial permeability transition pore
ATP: Adenosine triphosphate
ERK1/2: Extracellular signal-regulated kinase1/2
pERK1/2: Phosphorylated ERK1/2
MFN1: Mitofusin1
MFN2: Mitofusin2
DRP1: Dynamin-related protein1
pDRP1 ser616: Phosphorylated DRP1 ser616
FIS1: Fission-1
FBS: Fetal bovine serum
DAPI: 2-(4-Amidinophenyl)-6-indolecarbamidine dihydrochloride
