# Supplementary material for: Conditioned medium from induced pluripotent stem cell-derived mesenchymal stem cells accelerates cutaneous wound healing through enhanced angiogenesis
Source: Stem Cell Res Ther. 2021 May 20;12:295. doi: 10.1186/s13287-021-02366-x (PMC8139053; doi:10.1186/s13287-021-02366-x)
Supplement: Supplementary file 1 — Additional file 1. [file 13287_2021_2366_MOESM1_ESM.zip › supplementary Figure V1 without date-paper version V1.pptx]

## Slide 1
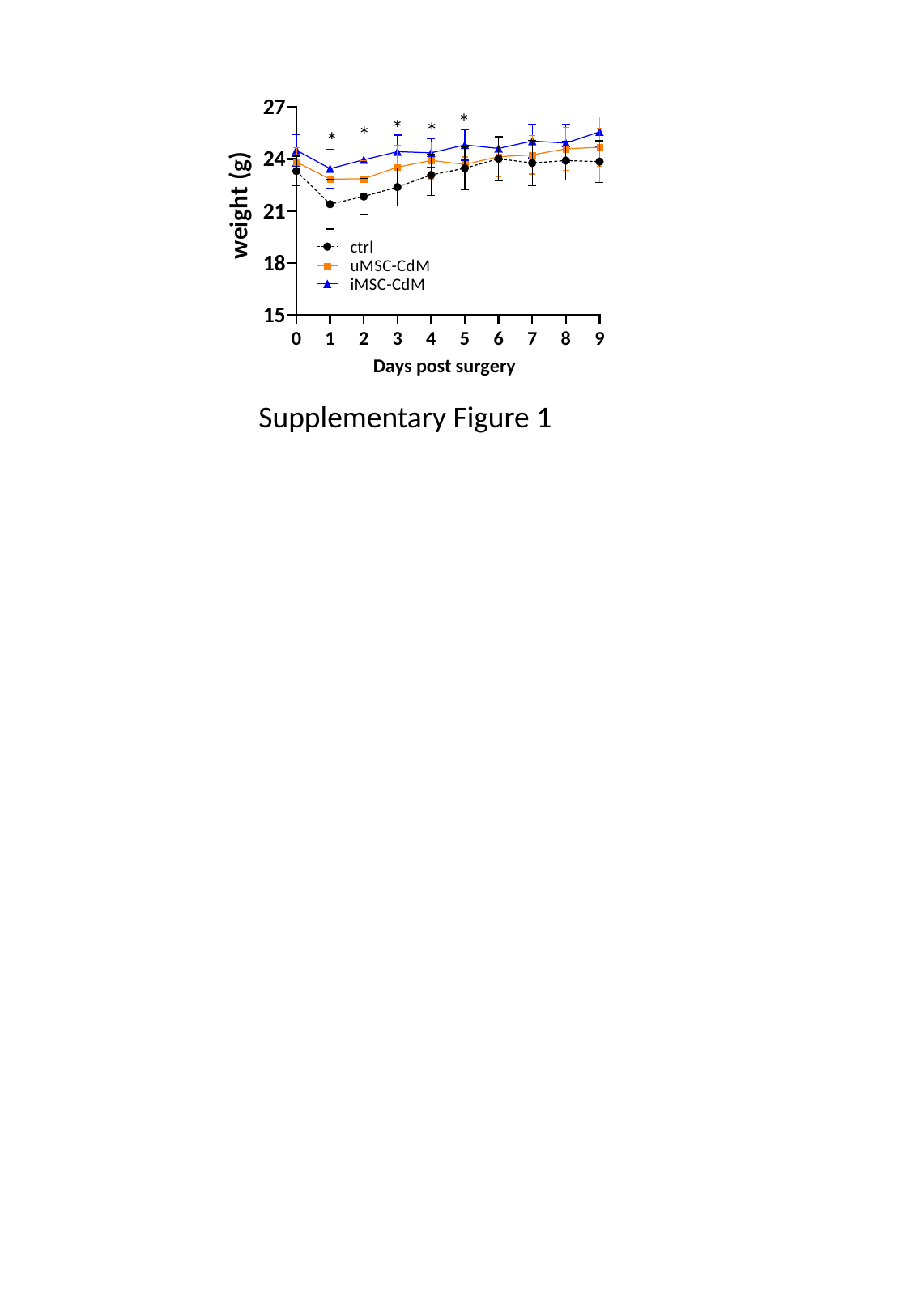

*
*
*
*
*
Supplementary Figure 1

## Slide 2
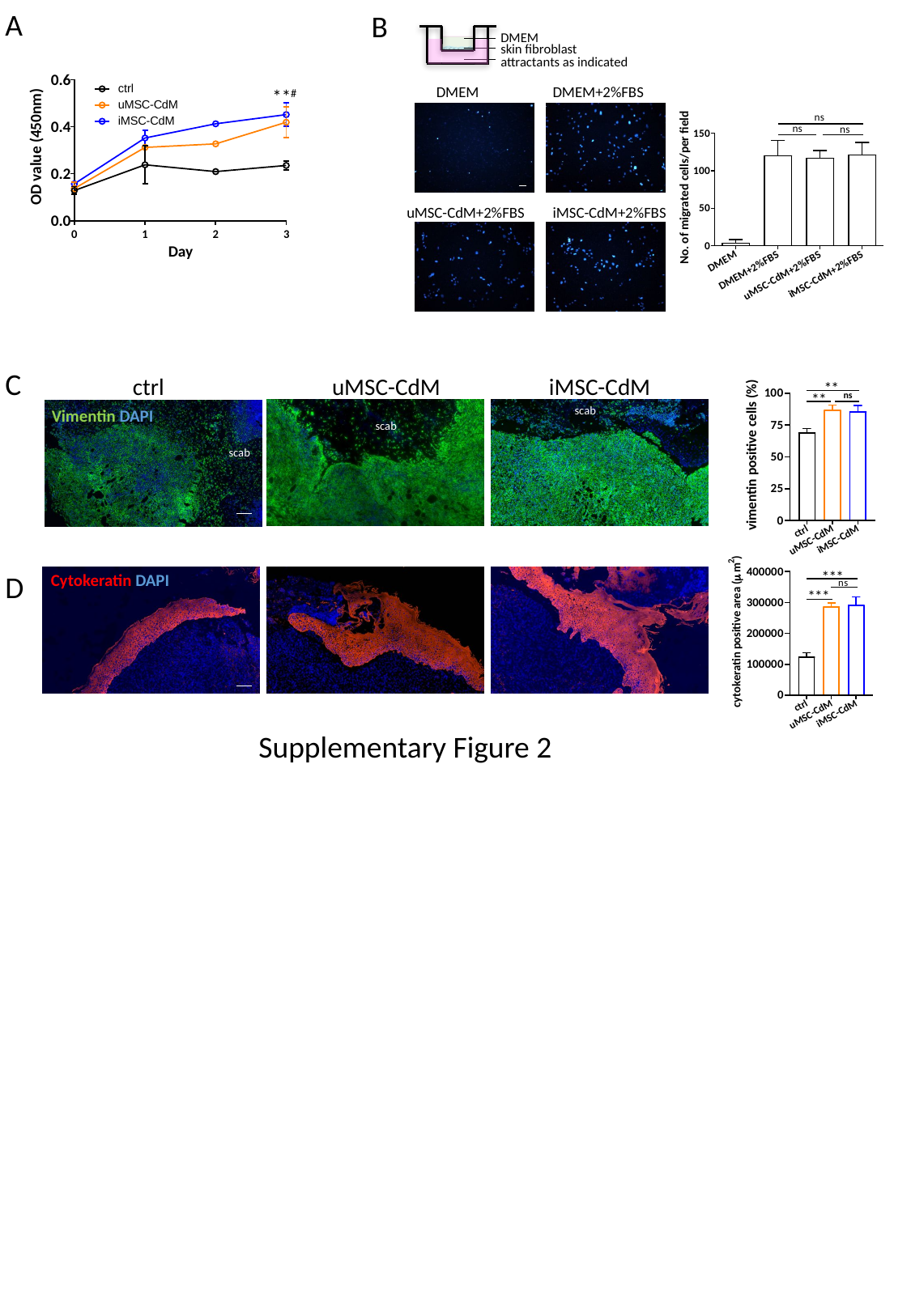

A
B
DMEM
skin fibroblast
attractants as indicated
DMEM
DMEM+2%FBS
#
**
uMSC-CdM+2%FBS
iMSC-CdM+2%FBS
C
ctrl
uMSC-CdM
iMSC-CdM
scab
Vimentin DAPI
scab
scab
D
Cytokeratin DAPI
Supplementary Figure 2

## Slide 3
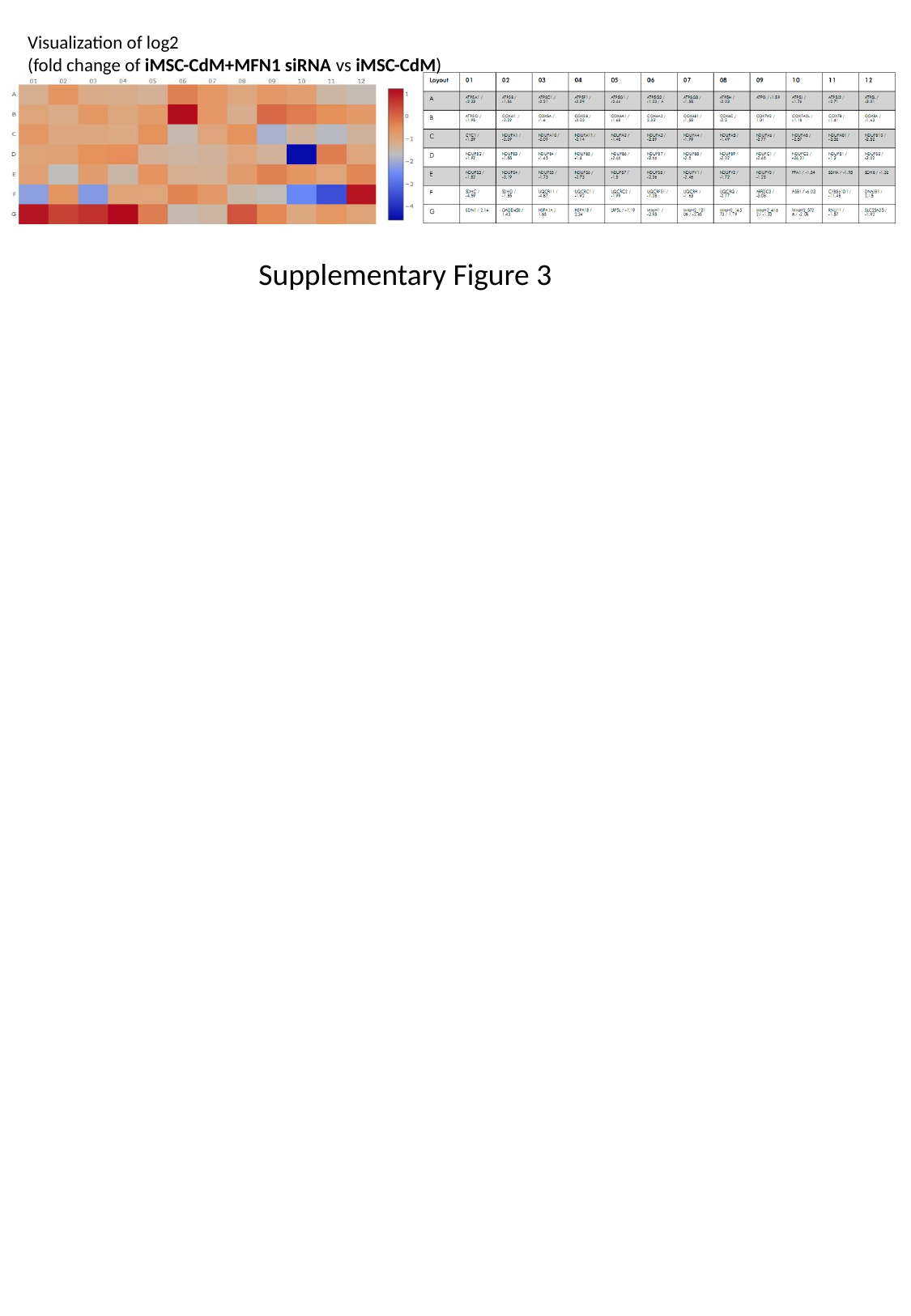

Visualization of log2
(fold change of iMSC-CdM+MFN1 siRNA vs iMSC-CdM)
Supplementary Figure 3
