# Supplementary material for: Conditioned medium from induced pluripotent stem cell-derived mesenchymal stem cells accelerates cutaneous wound healing through enhanced angiogenesis
Source: Stem Cell Res Ther. 2021 May 20;12:295. doi: 10.1186/s13287-021-02366-x (PMC8139053; doi:10.1186/s13287-021-02366-x)
Supplement: Supplementary file 1 — Additional file 1. [file 13287_2021_2366_MOESM1_ESM.zip › Supplementary information V2.docx]

**Supplementary information**

**Conditioned Medium From Induced Pluripotent Stem Cell-Derived Mesenchymal Stem Cells Accelerates Cutaneous Wound Healing through Mitigating Mitochondrial Dysfunction in Endothelial Cells**

**Running Head**

iPSC derived MSCs accelerates wound Healing

Xiaoting Liang^1,2^*, Fang Lin^2^*, Yue Ding^3^, Yuelin Zhang^4^, Mimi Li^2^, Xiaohui Zhou^2,^ Qingshu Meng^2^, Xiaoxue Ma^2^, Lu Wei^2^, Huimin Fan^2^#, Zhongmin Liu^1, 2, 5^#

1. Institute for Regenerative Medicine, Shanghai East Hospital, School of Life Sciences and Technology, Tongji University, Shanghai, P.R.China;
2. Research Center for Translational Medicine, Shanghai East Hospital, School of Medicine, Tongji University, Shanghai, P.R.China;
3. Department of Organ Transplantation, Changzheng Hospital, Second Military Medical University, Shanghai, P.R.China;
4. Department of Emergency, Guangdong General Hospital, Guangdong Academy of Medical Science, Guangzhou, P.R.China;
5. Department of Cardiovascular Surgery, Shanghai East Hospital, Tongji University School of Medicine, Shanghai, P.R.China.

*These authors contributed equally to this work.

Correspondence Information

Huimin Fan, MD. PhD

Department of Cardiovascular and Thoracic Surgery, Shanghai East Hospital, Tongji University School of Medicine, Shanghai, P.R.China

Email: frankfan@tongji.edu.cn

Zhongmin Liu, MD. PhD

Department of Cardiovascular and Thoracic Surgery, Shanghai East Hospital, Tongji University School of Medicine, Shanghai, P.R.China

liu.zhongmin@tongji.edu.cn

**Figure Legends**

**Supplementary Figure 1.** Daily body weight record after surgery. **P* < .05 iMSC-CdM vs control group.

**Supplementary Figure 2. Effects of uMSC-CdM/iMSC-CdM on fibroblasts and keratinocytes.** A) Skin fibroblasts proliferation was determined by Cell Counting Kit–8 assay. #*P* < .05 uMSC-CdM vs control group; ***P* < .01 iMSC-CdM vs control group. B) Experimental setting, representative images and quantification of transwell assay. C) Representative fluorescence images and quantification of vimentin (fibroblast marker). D) Representative fluorescence images and quantification of cytokeratin (keratinocyte marker). Scale bar = 100 µm. ns, non-signiﬁcance, ***P* < .01, ****P* < .001,

**Supplementary Figure 3.** Heat map of mitochondrial energy metabolism PCR array.
